# Structure-Guided Identification of a Nonhuman Morbillivirus with Zoonotic Potential

**DOI:** 10.1128/JVI.01248-18

**Published:** 2018-11-12

**Authors:** Nurshariza Abdullah, James T. Kelly, Stephen C. Graham, Jamie Birch, Daniel Gonçalves-Carneiro, Tim Mitchell, Robin N. Thompson, Katrina A. Lythgoe, Nicola Logan, Margaret J. Hosie, Vassiliy N. Bavro, Brian J. Willett, Michael P. Heaton, Dalan Bailey

**Affiliations:** a The Pirbright Institute, Surrey, United Kingdom; b Department of Pathology, University of Cambridge, Cambridge, United Kingdom; c The University of Birmingham, Birmingham, United Kingdom; d Department of Zoology, University of Oxford, Oxford, United Kingdom; e Mathematical Institute, University of Oxford, Oxford, United Kingdom; f Christ Church, University of Oxford, Oxford, United Kingdom; g Big Data Institute, University of Oxford, Oxford, United Kingdom; h MRC University of Glasgow Centre for Virus Research, Glasgow, United Kingdom; i School of Biological Sciences, University of Essex, Colchester, United Kingdom; j U.S. Meat Animal Research Center, Agricultural Research Service, U.S. Department of Agriculture, Clay Center, Nebraska, USA; University of Kentucky College of Medicine

**Keywords:** PPRV, host range, measles, morbillivirus, paramyxovirus, zoonoses

## Abstract

Morbilliviruses infect a broad range of mammalian hosts, including ruminants, carnivores, and humans. The recent eradication of rinderpest virus (RPV) and the active campaigns for eradication of the human-specific measles virus (MeV) have raised significant concerns that the remaining morbilliviruses may emerge in so-called vacated ecological niches. Seeking to assess the zoonotic potential of nonhuman morbilliviruses within human populations, we found that peste des petits ruminants virus (PPRV)—the small-ruminant morbillivirus—is restricted at the point of entry into human cells due to deficient interactions with human SLAMF1—the immune cell receptor for morbilliviruses. Using a structure-guided approach, we characterized a single amino acid change, mapping to the receptor-binding domain in the PPRV hemagglutinin (H) protein, which overcomes this restriction. The same mutation allowed escape from some cross-protective, human patient, anti-MeV antibodies, raising concerns that PPRV is a pathogen with zoonotic potential. Analysis of natural variation within human and ovine SLAMF1 also identified polymorphisms that could correlate with disease resistance. Finally, the mechanistic nature of the PPRV restriction was also investigated, identifying charge incompatibility and steric hindrance between PPRV H and human SLAMF1 proteins. Importantly, this research was performed entirely using surrogate virus entry assays, negating the requirement for *in situ* derivation of a human-tropic PPRV and illustrating alternative strategies for identifying gain-of-function mutations in viral pathogens.

**IMPORTANCE** A significant proportion of viral pandemics occur following zoonotic transmission events, where animal-associated viruses jump species into human populations. In order to provide forewarnings of the emergence of these viruses, it is necessary to develop a better understanding of what determines virus host range, often at the genetic and structural levels. In this study, we demonstrated that the small-ruminant morbillivirus, a close relative of measles, is unable to use human receptors to enter cells; however, a change of a single amino acid in the virus is sufficient to overcome this restriction. This information will be important for monitoring this virus’s evolution in the field. Of note, this study was undertaken *in vitro*, without generation of a fully infectious virus with this phenotype.

## Abstract (precis)

A significant proportion of viral pandemics occur following zoonotic transmission events, where animal-associated viruses jump species into human populations. In order to provide forewarnings of the emergence of these viruses, it is necessary to develop a better understanding of what determines virus host range, often at the genetic and structural levels. In this study, we demonstrated that the small-ruminant morbillivirus, a close relative of measles, is unable to use human receptors to enter cells; however, a change of a single amino acid in the virus is sufficient to overcome this restriction. This information will be important for monitoring this virus’s evolution in the field. Of note, this study was undertaken *in vitro*, without generation of a fully infectious virus with this phenotype.

## INTRODUCTION

Morbilliviruses remain significant causes of animal and human disease in populations that are fundamental to our continued medical, economic, and ecological security. Measles virus (MeV) kills nearly 100,000 people each year despite the availability of efficacious vaccines (1), while canine distemper virus (CDV) has caused outbreaks in endangered lion, tiger, and primate populations (2, 3). Globally, over 1 billion sheep and goats, representing approximately 80% of the world’s small ruminants, are at risk from peste des petits ruminants virus (PPRV) infection (4). In addition, aquatic mammals such as whales, dolphins, porpoises, and seals are also target species for morbilliviruses; recent epidemics of phocine distemper virus (PDV) and cetacean morbillivirus (CeMV) in northwestern Europe have resulted in large fluctuations in herd immunity in these wildlife populations (5).

In the developing world, PPRV represents one of the key challenges to sustainable small-ruminant agriculture (6) where high-mortality epidemics, combined with longer-term endemicity, combine to undermine subsistence farming (7, 8). The distribution of this virus is also widening, with recent outbreaks in Morocco, Georgia, Mongolia, and Bulgaria. Collectively, the economic losses associated with PPRV are now estimated to be $1.45 to $2.1 billion per year (9, 10). Given these factors and the genetic similarity of morbilliviruses and their life cycles, examining the “zoonotic potential” of nonhuman morbilliviruses is both timely and warranted.

**FIG 1:**
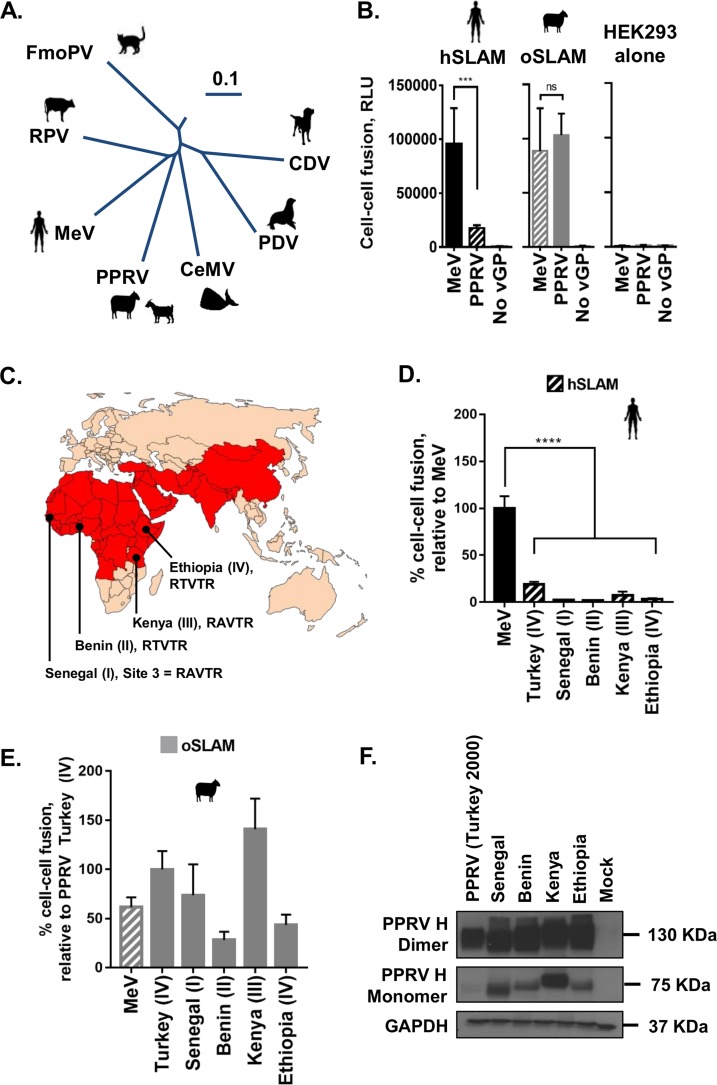
PPRV glycoprotein-mediated fusion is restricted with the human SLAMF1 receptor. (A) The established mammalian morbilliviruses (phylogenetic tree assembled from complete genome sequences using Vector Nti; 0.1 scale bar; nucleotide substitutions). FmoPV, feline morbillivirus. (B) MeV cell-cell fusion is efficient when either hSLAM (black) (left panel) or oSLAM (gray) (middle panel) is present on target cells; however, PPRV is restricted to efficient fusion with oSLAM only. Minimal fusion is seen with HEK293 cells alone (right panel). Raw *Renilla* luciferase assay data are shown (RLU, relative light units). Noncognate virus-receptor interactions are indicated with patterned shading. (C) The current distribution of PPRV according to the World Organization for Animal Health (OIE) and the Food and Agriculture Organization for the United Nations (FAO). The countries highlighted in red have ongoing (or previously had) outbreaks of PPRV. Additional representative isolates of PPRV or genetic lineages I to IV from the indicated countries (Senegal, Benin, Kenya, and Ethiopia) were also used in this study to confirm the hSLAM restriction. The amino acid sequences of the PPRV H RBD at site 3 (aa 191 to 195) from these additional isolates (RAVTR and RTVTR) are shown. (D and E) All PPRV H proteins tested showed similar restrictions with respect to hSLAM-mediated cell-cell fusion. Results are expressed relative to cognate virus-host interactions, i.e., MeV-hSLAM (D) or PPRV (Turkey; lineage IV)-oSLAM (E). Noncognate virus-receptor interactions are indicated with patterned shading. (F) Western blot analysis of the various PPRV H proteins, from distinct lineages, expressed in the cell-cell fusion assays; both monomeric H and dimeric H were detected using an antibody targeting the cytoplasmic tail of H (H-cyt). Graphs denote the mean activity levels determined for >4 biological replicates, with error bars denoting standard deviations. Statistical analysis: one-way analysis of variance (ANOVA) with Dunnett’s multiple-comparison test (*, *P* = <0.05; **, *P* = <0.01; ***, *P* = <0.005; ****, *P* = <0.001; ns, nonsignificant).

A critical feature of morbilliviruses is that they use the same proteinaceous receptors to enter host cells, namely, signaling lymphocytic activation molecule F1 (SLAMF1) on immune cells and Nectin-4 on polarized epithelial cells (11, 12). The current model for morbillivirus pathogenesis is that SLAMF1 is the key “entry receptor” and that immune cells in the upper respiratory tract are the first to become infected. These migrate to local lymph nodes, initiating robust replication and the development of cell-associated viremia. At this stage, Nectin-4, which is expressed basolaterally on polarized epithelia in various tissues, serves as the “exit receptor” facilitating virus escape, i.e., following apical shedding into the lumen of the lung (12). The universal use of SLAMF1 and Nectin-4 as morbillivirus receptors is long established (especially for SLAMF1 [13]) and has been the source of intense investigation with regard to its role in determining host range (14). This conserved receptor usage is likely the result of direct evolution from a single common ancestor. Although the host tropism of this ancestral virus is unknown, a specific relationship between MeV and rinderpest virus (RPV) has been identified (Fig. 1A) and there is supporting genetic evidence that MeV emerged in humans following a zoonotic transmission event, perhaps during the domestication of cattle (15). The currently established morbilliviruses retain a great deal of genetic similarity (63% to 66% at the genome level [16]) and have been used interchangeably during vaccination, since their related antigenicity can, in some contexts, provide effective cross-protective immunity (17). The exact genetic determinants of morbillivirus host range remain relatively poorly characterized, however, and it is unclear which stages of the viral life cycle contribute to the restricted disease host range observed in the field or clinic. Establishing the nature of these barriers is therefore fundamental to the continued control of these important viruses. For instance, eradication of RPV led to cessation of vaccination, but, as a result, there are now realistic fears that PPRV or CDV could spill over into the global population of 1.5 billion immunologically naive cattle (18).

We posited that viral entry may represent one such barrier and may influence the potential zoonotic transmission of nonhuman morbilliviruses into human populations. The prevalent strategy for investigating this restriction is direct examination of human receptor usage using field isolates of nonhuman morbilliviruses, e.g., monitoring PPRV infection of human SLAMF1-bearing cells. Indeed, this approach was previously employed to demonstrate that a CDV isolate could not efficiently use human SLAMF1 to enter Vero cells (19). Those authors subsequently demonstrated that repeated passage in the presence of this receptor could overcome this restriction. In fact, the low-fidelity RNA-dependent RNA polymerases of RNA viruses have frequently been exploited in this context to identify genetic mutations that support zoonotic transmission, e.g., influenza virus H5N1 (20, 21). These approaches, which normally involve the application of a strong selection pressure through serial passaging of viruses *in vitro* or *in vivo*, are broadly referred to as classical gain-of-function (GOF) experiments and have provided important epidemiological information together with an improved understanding of pathogen biology. However, the accompanying *in situ* creation of GOF viruses has caused controversy, in particular because of biosafety concerns regarding the accidental or purposeful release of laboratory-derived pathogens with zoonotic potential (22, 23).

In light of these concerns, we hypothesized that, by building on our existing structural and mechanistic understanding of morbillivirus attachment (24), host receptors (12, 25, 26), and particle entry/fusion (27), we could reliably predict GOF mutants *a posteriori*, without *in situ* derivation of a GOF morbillivirus. The following research data confirmed that these approaches are valid and, further, that zoonotic restrictions do exist at the point of entry of nonhuman morbillivirus, in this case, PPRV, into human cells. As observed in the related CDV study (19), these restrictions are easily overcome by small amino acid substitutions within the receptor-binding domain (RBD); however, we were also able to demonstrate that these mutations have concomitant effects on antibody (Ab)-mediated neutralization. In addition, using our novel approaches and assays, we were able to arrive at a mechanistic understanding of this restriction at the structural level, all in the absence of classical GOF experiments.

## RESULTS

### PPRV glycoprotein-mediated fusion is restricted with the human SLAMF1 receptor.

Seeking to address whether receptor usage represents a critical barrier to the zoonotic transmission of PPRV, we focused on viral interactions with SLAMF1. The H-SLAMF1 protein-protein interaction represents the first step in virus attachment, prior to activation of the fusion (F) protein and virus-cell membrane fusion. Taking advantage of an adapted bifluorescence reporter (28), we used a quantitative cell-cell fusion assay to compare the levels of receptor usage by the macromolecular FH complexes of MeV and PPRV. While MeV induced similar levels of fusion with human SLAMF1 and ovine SLAMF1 (hSLAM and oSLAM, respectively), PPRV-induced fusion was severely restricted when paired with the hSLAM noncognate receptor (Fig. 1B). This restriction was subsequently confirmed with H proteins representing field isolates from all four genetic lineages of PPRV (Fig. 1C to F).

### Sequence variation within RBD site 3 may determine virus host range.

**FIG 2:**
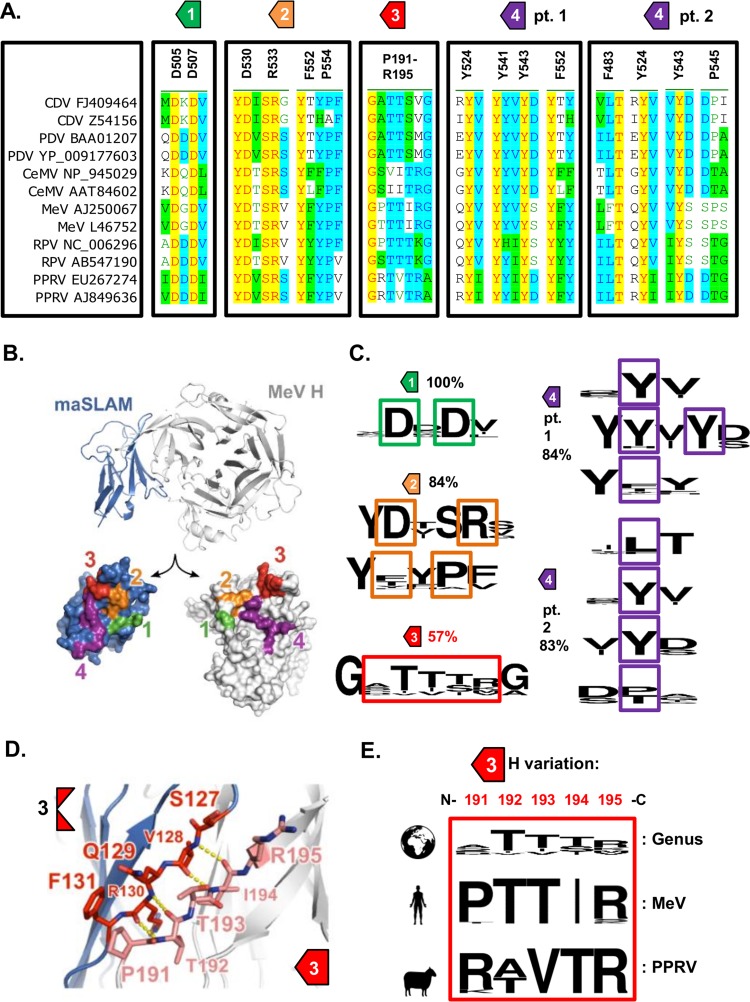
Sequence variation within RBD site 3 may determine virus host range. (A) Multiple alignments of various morbillivirus H proteins, focusing on the 4 sites that constitute the RBD. The surrounding sequences are shown for clarity, although the exact amino acid positions and sequences are indicated (relative to the MeV isolate from Dublin). Of note, a number of specific amino acid residues were found in more than one site (Y524 and F552). The PPRV reference sequence used in this study is PPRV AJ849636. The lapinized RPV strain is AB547190. (B) Structure of measles virus H (MeV H) in complex with marmoset SLAMF1 (maSLAM) receptor (PDB 3ALX) (24). (Top) The complex is shown as ribbons. (Bottom) The interaction interfaces between MeV and maSLAM are shown as molecular surfaces that are labeled 1 to 4 and colored as defined by Hashiguchi et al. (24). (C) Multiple alignments of various morbillivirus H proteins, focusing on the 4 sites that constitute the RBD. The surrounding sequences are shown for clarity, although the exact amino acid positions and sequences are highlighted. Conservation within the separate sites was analyzed using the WebLogo online server. The overall variation detected (given as percent identity) within each site is also indicated. The sequences used for this analysis are provided in Data Set S1 together with the relative amino acid positions. (D) MeV H:maSLAM interaction region 3 comprises an interaction between adjacent anti-parallel β-sheets. The ribbon representation is colored as described for panel B, with selected residues shown as sticks (MeV H carbon atoms in pink, maSLAM carbon atoms in red) and selected backbone hydrogen bonds shown (yellow dots). (E) Morbillivirus interspecies amino acid variability within site 3 (aa 191 to 195; N-C amino acid termini are indicated) is high (comparative sequence alignment is indicated at the top of the panel); however, intraspecies variability is markedly lower (analysis of a complete spectrum of circulating MeV and PPRV genotypes is indicated at the bottom of the panel—see Data Set S1).

The structure of a chimeric protein representing the complex between MeV H and marmoset SLAM (maSLAM) (84% sequence identity with human SLAM) has been solved (24). Examination of genus-wide sequence conservation within the four identified interaction motifs (sites 1 to 4; Fig. 2A and B) of the RBD (24) revealed significant variation (<80% sequence identity) only within site 3 (Fig. 2C; see also Data Set S1 in the supplemental material) and an interaction between antiparallel intermolecular β-strands of SLAMF1 and H (Fig. 2D). A more in-depth analysis of intraspecies variability within this site, using representative isolates from all genetic lineages, identified a contrasting pattern, with conservation of H sequences _191_PTTIR_195_ and _191_R(A/T)VTR_195_ in various strains of MeV and PPRV, respectively (Fig. 2E; see also Data Set S1).

### Minor changes to the PPRV H RBD overcome species-specific restrictions.

**FIG 3:**
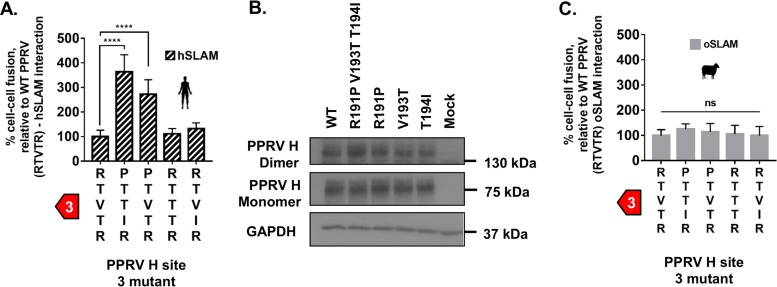
Minor changes to the PPRV H RBD overcome species-specific restrictions. (A) A chimeric PPRV H containing the site 3 amino acid sequence of MeV (RTVTR—PTTIR) is no longer restricted by hSLAM activity in the cell-cell fusion assay. Results are expressed relative to wild-type (WT) PPRV H and hSLAM interactions (see Fig. 1B). (B) Western blot analysis of the WT and mutant PPRV H expressed in effector cells shows equivalent expression levels of both monomeric and dimeric H protein. (C) MeV chimeric mutations within PPRV H site 3 have a neutral effect on oSLAM-dependent fusion. Results are expressed relative to WT PPRV H and oSLAM interactions. In all panels, graphs denote the mean activity from biological replicates, with error bars denoting standard deviations. Statistical analysis: one-way ANOVA with Dunnett’s multiple comparisons tests (*, *P* = <0.05; **, *P* = <0.01; ***, *P* = <0.005; ****, *P* = <0.001; ns, nonsignificant).

Building on the hypothesis that variation at site 3 could be an important genetic determinant of host range, we generated a chimeric PPRV H protein containing the PTTIR sequence at this site (PPRV^MeV^ H) and examined its capacity to support cell-cell fusion. This mutant had a significant GOF phenotype with hSLAM (Fig. 3A), a change dependent on a single R/P change at amino acid (aa) position 191 (Fig. 3A; R191P). In addition, substitutions within site 3 did not lead to clear changes in protein stability (Fig. 3B) or significantly alter oSLAM-related fusion (Fig. 3C), identifying these mutations as effectively neutral with respect to PPRV infection in sheep, should they be acquired naturally.

### Restriction and GOF phenotypes are recapitulated in a separate model of viral entry.

**FIG 4:**
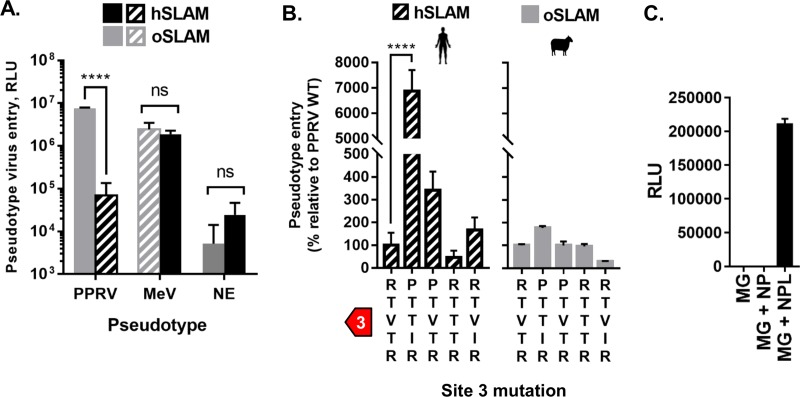
Restriction and GOF phenotypes are recapitulated in a separate model of viral entry. (A) Equivalent levels of PPRV H hSLAM restriction were observed using a PPRV-pseudotyped (PP) HIV-1-based entry assay. The graph denotes the mean activity from >4 biological replicates, with error bars denoting standard deviations. Noncognate virus-receptor interactions are indicated with patterned shading. Statistical analysis: Two-way ANOVA with Sidak’s multiple-comparison test (****, *P* = <0.001; ns, not significant). NE, nonenveloped control pseudotypes. (B) PPRV PPs bearing PPRV^MeV^ H protein chimeras overcome the hSLAM restriction, specifically, the full site 3 chimera (PTTIR) and the single R191P mutation, while having minimal effects on oSLAM-mediated fusion. Results are expressed as percent change relative to PPRV WT H (unmutated) PP entry. Graphs denote the mean activity from >4 biological replicates, with error bars denoting standard deviations. Noncognate virus-receptor interactions are indicated with patterned shading. Statistical analysis: one-way ANOVA with Dunnett’s multiple-comparison test (*, *P* = <0.05; **, *P* = <0.01; ***, *P* = <0.005; ****, *P* = <0.001; ns, nonsignificant). (C) A PPRV minigenome, supported by *trans*-expression of PPRV N, P, and L proteins, is functional in human A549 cells. Experiments were performed in triplicate. Error bars denote standard deviations; luciferase assays were normalized against untransfected cells.

Using a related entry assay, i.e., pseudotyping of PPRV FH glycoproteins on the surface of replication-incompetent HIV-1 particles, we confirmed both the original restriction (Fig. 4A) and the GOF characteristics of the chimeric PPRV^MeV^ H (Fig. 4B). Unlike the results seen with the primary H-SLAMF1 interaction interface, substitution of residues at the interfaces between adjacent molecules within the tetrameric H (form II [24]) had only modest effects on host-cell fusion in the presence of either cognate or noncognate SLAM receptors (data not shown). Combined, these data indicate that small changes within the RBD of PPRV H are sufficient to alleviate host barriers to zoonotic transmission. Finally, we used a minigenome assay to confirm that the PPRV RNA replication machinery is functional in human cells, indicating that entry alone may be the critical barrier to PPRV replication in human cells (Fig. 4C). Although our observations were made using surrogate models of morbillivirus entry, cell-cell fusion, and RNA replication, similar assays have been shown to faithfully recapitulate native viral activity (28–30).

### Random mutagenesis of PPRV H R191 identifies numerous GOF mutations.

**FIG 5:**
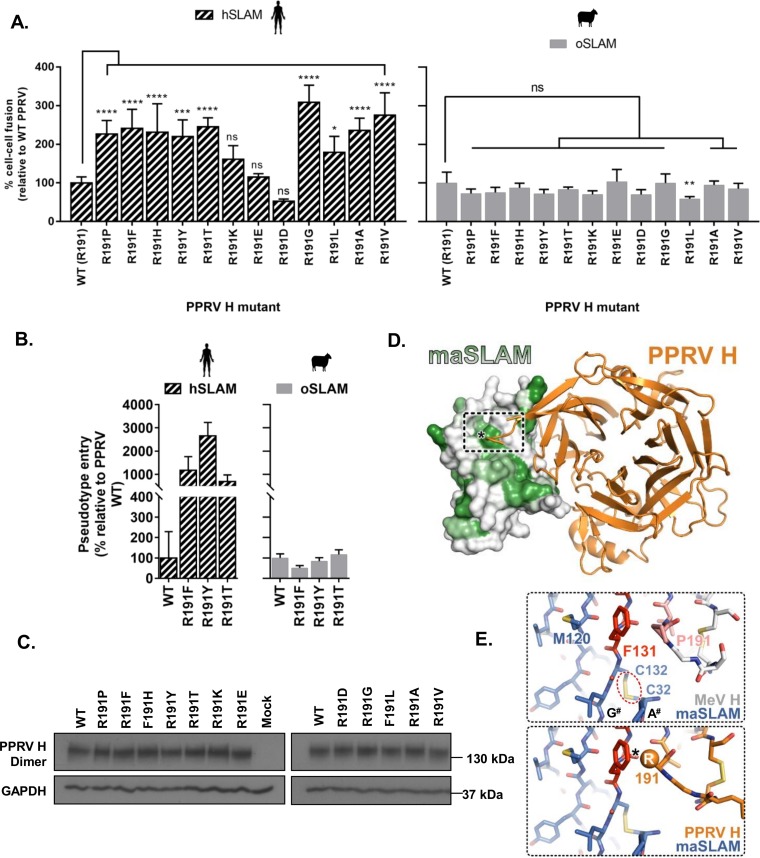
Random mutagenesis of PPRV H R191 identifies numerous GOF mutations. (A) Using the standard cell-cell fusion assay, random mutagenesis of the arginine (R) residue in PPRV H (R191) revealed that multiple amino acid changes are sufficient to overcome the PPRV-hSLAM restriction (diagonally striped bars). The effects of these amino acid changes on oSLAM-dependent fusion are also indicated (gray bars). All results are expressed relative to PPRV WT H (unmutated) interactions with hSLAM and oSLAM (left and right panels, respectively). (B) Similar results were observed using a subset of these mutants and the PPRV-pseudotyped (PP) HIV-1-based entry assay; all results are expressed as percent change relative to PPRV WT (unmutated) H PP entry. Left panel, hSLAM; right panel, oSLAM. (C) Various mutations at PPRV H amino acid position 191 do not affect the relative stability of this protein. kDa size markers refer to SDS-PAGE ladder positions. (D) Model of PPRV H in complex with maSLAM. The maSLAM surface is colored according to residue hydrophobicity, from white (polar) to green (hydrophobic). (E) H residue 191 interacts with conserved SLAM residue 131. In the maSLAM-plus-MeV H complex (top), the prolidyl ring of P191 forms a stacking interaction with the hydrophobic F131 side chain. The adjacent disulfide bond that connects the A and G strands (A^#^ and G^#^) of the maSLAM Ig domain is highlighted. The presence of the bulky charged residue arginine at this location in PRRV H (bottom) would likely interfere with complex formation. Residues in MeV H:maSLAM interaction region 3 are colored as described for Fig. 2D. In all panels, graphs denote the mean activity from >4 biological replicates, with error bars denoting standard deviations. Statistical analysis: one-way ANOVA with Dunnett’s multiple-comparison test (*, *P* = <0.05; **, *P* = <0.01; ***, *P* = <0.005; ****, *P* = <0.001).

Our *in vitro* receptor usage assays permit rapid screening of H variants for GOF phenotypes without the need for recombinant virus handling under conditions of high-level containment. To expand our analysis of PPRV GOF mutations, we used degenerate primers to generate additional R191 mutants, all of which (except R191K, R191E, and R191D) were able to overcome the PPRV hSLAM restriction in cell-cell fusion at significant levels while having a negligible impact on oSLAM-mediated fusion (Fig. 5A). Similar results were observed with a subset of these mutants adapted for the pseudotype viral entry assay (Fig. 5B). In addition, the relative stabilities of these proteins were again apparently unaffected by mutation at position 191 (Fig. 5C). A model of PPRV H in complex with maSLAM showed residue 191 to be in close proximity to a hydrophobic region of the SLAMF1 surface (Fig. 5D) centered on conserved residue F131 (Fig. 5E). Our analysis suggests that the PPRV hSLAM restriction is caused by a combination of charge and steric hindrance between PPRV H R191 and this region, thus providing a mechanistic basis for this restriction. It is likely that oSLAM has a distinct conformation in this region that accommodates PPRV H binding, as oSLAM lacks the disulfide bond linking the A and G strands that is present in hSLAM (C32 to C132; Fig. 5E) and has an extra residue in the A strand compared to hSLAM (Data Set S1).

### Minor variations in mammalian SLAMF1 proteins determine morbillivirus host range.

**FIG 6:**
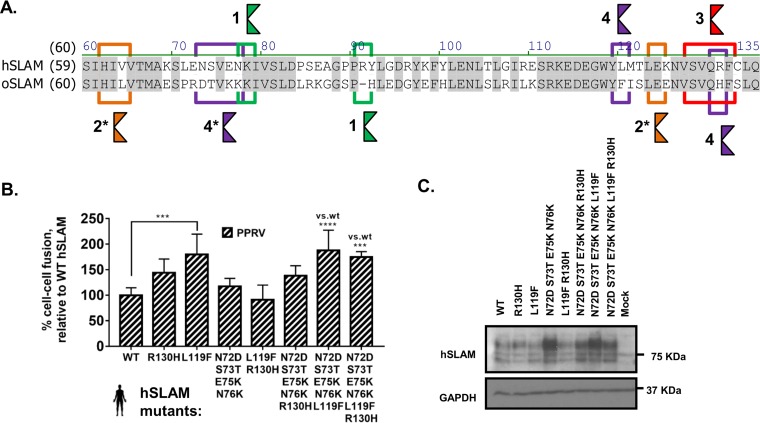
Minor variations in mammalian SLAMF1 proteins determine morbillivirus host range. (A) An amino acid alignment of the HBS of hSLAM and oSLAM highlights variations between the two proteins in sites 1 to 4. The asterisks (*) indicate that analysis of site 4 was extended to include S73 and N76. (B) Stepwise mutation of the hSLAM sequence to generate a site 3 or site 4 oSLAM chimera is sufficient to overcome the PPRV-hSLAM restriction. Results are expressed relative to WT PPRV H protein interactions with the unmutated WT hSLAM receptor. (C) Western blot analysis of the WT and mutant hSLAM sequences expressed in target cells (antibody, SLAM [N-19], Santa Cruz sc-1334). Graphs denote the mean activity from >4 biological replicates, with error bars denoting standard deviations. Statistical analysis: one-way ANOVA with Dunnett’s multiple-comparison test (*, *P* = <0.05; **, *P* = <0.01; ***, *P* = <0.005; ****, *P* = <0.001; ns, nonsignificant).

To examine the host-specific restrictions to PPRV entry, targeted mutagenesis of hSLAM was performed by substituting oSLAM residues within the four motifs constituting the H-binding site (HBS) in SLAMF1 (Fig. 6A). Generation of an hSLAM^oSLAM^ chimera in this context was sufficient to partially overcome the PPRV-hSLAM restriction, confirming the importance of these four motifs in general, and of the single L119F substitution in particular, for virus attachment (Fig. 6B). Antibody-mediated recognition of this protein, however, appeared to be modified (Fig. 6C), possibly due to modification of the epitope—a finding supported by the results of flow cytometry performed with a separate commercial antibody (mouse anti-human CD150, clone A12; BD Biosciences) (data not shown). In conclusion, the PPRV-hSLAM restriction barrier is dependent on the compatibility of several key interacting side chains within the HBSs of hSLAM and oSLAM.

### Host-encoded variability in SLAMF1 affects receptor usage by morbilliviruses.

**FIG 7:**
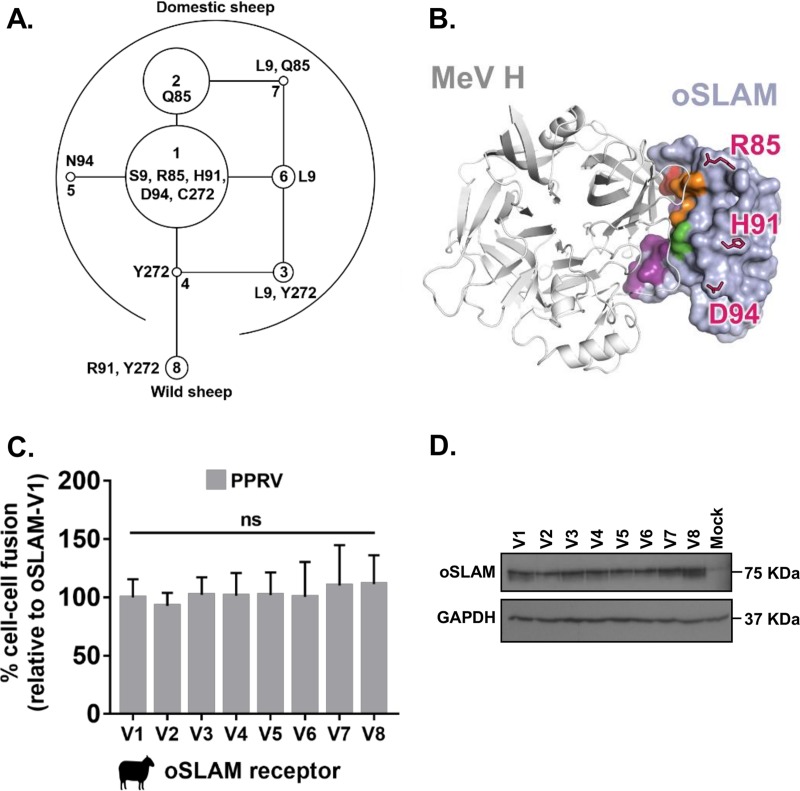
Host variation in oSLAM is not associated with altered PPRV-glycoprotein mediated fusion. (A) Amino acid variants encoded within the ovine *SLAMF1* gene locus were characterized in a diverse set of 171 sheep with available genome sequences. From these data, a rooted maximum parsimony phylogenetic tree of the haplotype-phased protein variants was constructed. Each node in the tree represents a different, naturally occurring protein isoform; the isoforms differ by single amino acids. The areas of the circles are proportional to the variant frequencies (see Data Set S1). (B) Model of MeV H (white ribbons) in complex with oSLAM (light blue molecular surface), showing that the ovine SNPs (pink sticks) lie outside the likely HBS (residues equivalent to maSLAM HBS are colored as described for Fig. 2B). (C) Variation in oSLAM does not markedly affect the cell-cell fusion potential of PPRV F and H proteins. Results are expressed relative to the dominant variant of oSLAM (V1; S_9_R_85_H_91_D_94_C_272_). (D) Western blot analysis of the eight variant oSLAMs (V1 to V8) expressed in target cells (antibody: His tag). The graph denotes the mean activity from >4 biological replicates, with error bars denoting standard deviations. Statistical analysis: one-way ANOVA with Dunnett’s multiple-comparison test (*, *P* = <0.05; **, *P* = <0.01; ***, *P* = <0.005; ****, *P* = <0.001).

**FIG 8:**
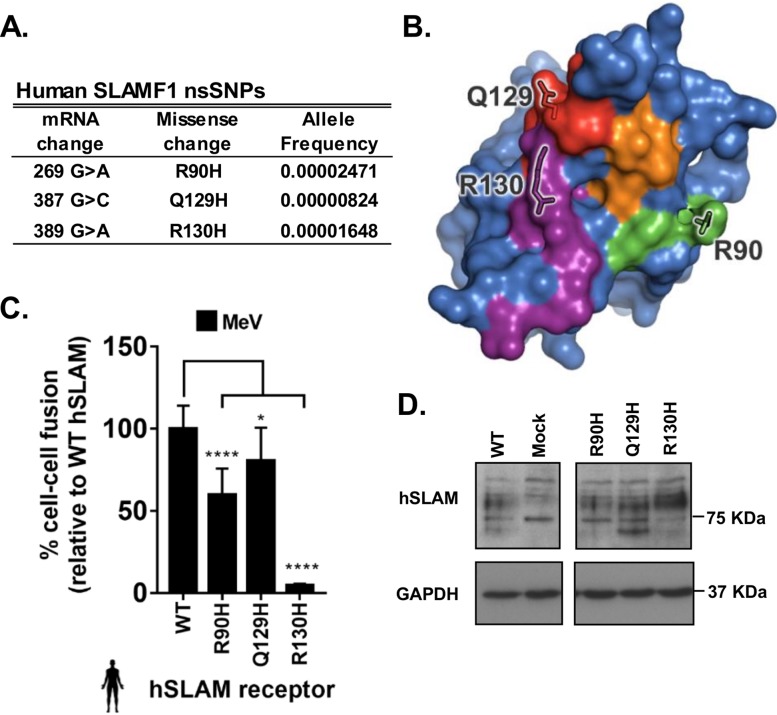
Minor-frequency nsSNPs within the hSLAM HBS are associated with altered MeV-glycoprotein mediated fusion. (A) Allele frequencies of human SLAMF1 nsSNPs within the HBS of hSLAM. (B) Residues equivalent to nsSNPs found within the publicly available EXAC database and the hSLAM HBS are shown on the molecular surface of maSLAM (HBS regions are colored as described for Fig. 2B). (C) Variation within the hSLAM HBS reduced MeV-induced cell-cell fusion. Results are expressed relative to the WT hSLAM amino acid sequence (UniProt accession no. Q13291). (D) Western blot analysis of the three variant hSLAMs (and of the dominant sequence [WT]) expressed in target cells. The graph denotes the mean activity from >4 biological replicates, with error bars denoting standard deviations. Statistical analysis: one-way ANOVA with Dunnett’s multiple-comparison test (*, *P* = <0.05; **, *P* = <0.01; ***, *P* = <0.005; ****, *P* = <0.001).

Since the role of host genetic variability in morbillivirus entry has not been fully characterized (11), especially with respect to nonsynonymous single nucleotide polymorphisms (nsSNPs) within SLAMF1, we generated variant SLAMF1 sequences for oSLAM and hSLAM, based on those known in sheep and human populations (31, 32), and examined their capacity to support PPRV- and MeV-induced fusion (Fig. 7 and 8). Ovine SLAMF1 polymorphisms mapped outside the HBS (Fig. 7A and B; see also Data Set S1) and therefore, unsurprisingly, did not affect PPRV-induced fusion, even when expressed in their biologically relevant context (haplotype variants 1 to 8) (Fig. 7C). There was, however, no marked difference between the variants with respect to protein stability (Fig. 7D). Due to the abundance (*n* = 104) of nsSNPs recorded for human SLAMF1 in the Exome Aggregation Consortium (ExAC) database (32), we targeted only nsSNPs mapping to the HBS (R90H, Q129H, and R130H) (Fig. 8A and B). All substitutions significantly reduced MeV-induced fusion (Fig. 8C), thus identifying naturally occurring and, potentially, resistance-associated polymorphisms. R90 sits within a multiresidue charge interaction network; the intermediary phenotype of the R90H substitution is likely due to removal of a salt bridge with D507, reducing the strength of these interactions and yet retaining charge compatibility. As noted by Hashiguchi et al. (24), MeV H residue R130 forms a salt bridge with MeV H residue E75 and plays a significant role in the H-SLAMF1 interaction, forming the centerpiece of a large interaction network. The complete loss in fusion conferred by the R130H substitution likely arises from removal of the salt bridge and disruption of this supporting network. In contrast, the marginal phenotype observed for the Q129H substitution likely arises from the predominately backbone-mediated nature of the interaction between β-sheets at site 3, with the Q129 side chain being on the side of the β-sheet opposite the primary interaction site (Fig. 2D). In contrast to the oSLAM variants, hSLAM SNPs within the RBD were detected in variable quantities following Western blotting (Fig. 8D), which is consistent with our previous findings indicating that the antibody epitope may be modified. Nevertheless, we believe that these specific changes may correlate with genetic polymorphisms that may confer resistance to disease.

### Cross-protective neutralization is affected by gain-of-function mutations within the PPRV H RBD.

**FIG 9:**
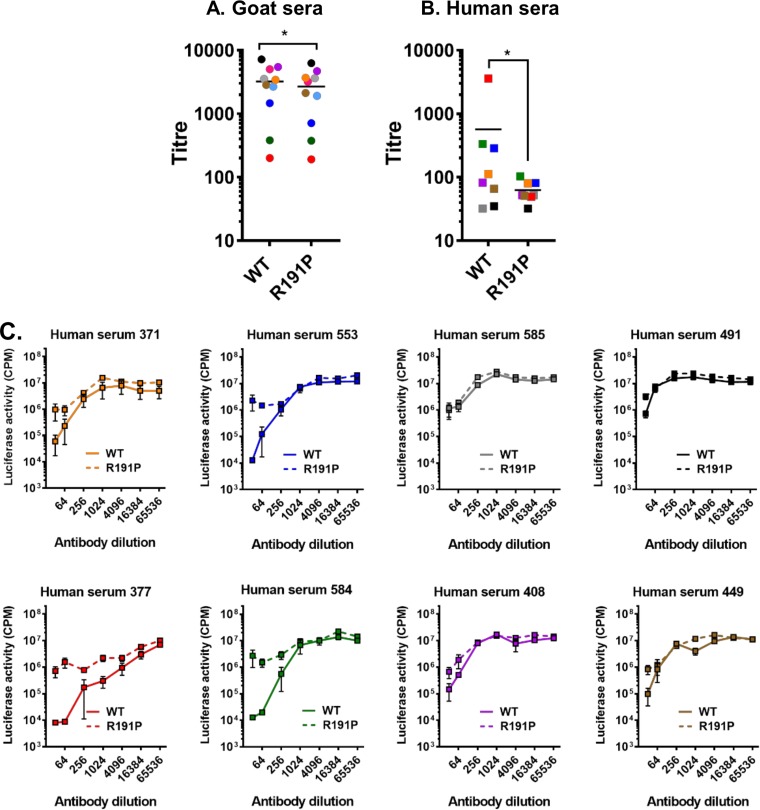
Cross-protective neutralization is affected by gain-of-function mutations within the PPRV H RBD. (A and B) Neutralization of WT and R191P-bearing PPRV PPs by sera from goats (PPRV specific; left panel; *n* = 10) (A) and humans (MeV specific, right panel; *n* = 8) (B). EC_90_ titers (color-matched by serum) are shown; error bars denote standard errors of the means. Antibody titers were calculated by interpolating the point at which there was a 90% reduction in luciferase activity (90% neutralization or 90% inhibitory concentration [EC_90_]). Statistical analysis was performed using the nonparametric Wilcoxon matched-pair signed rank test (*, *P* =<0.05). (C) In certain human serum samples, e.g., 377 and 584 (red and green squares, respectively), R191P confers a nAb escape phenotype to PPRV PPs. Surrogate VNT titrations were performed with individual MeV-specific human sera to calculate cross-protective titers against WT and R191P PPRV pseudotypes. Titrations were performed in triplicate on nonrestricted HEK293 canine SLAM cells, with error bars denoting standard errors of the means. The coloring of the lines within the graphs matches the sera used in the summary EC_90_ panel (i.e., panel B).

We next examined immune recognition of the isolated GOF mutants by the host. Morbillivirus infection or vaccination is known to elicit a strong neutralizing antibody (nAb) response to F and/or H which is critical to life-long immunity in vaccinated or convalescent hosts (33). The monoserotypic nature of morbilliviruses and their close genetic relationship also favor the development of cross-protective nAbs (34), which we theorized may protect against zoonotic transfer. Using a surrogate virus neutralization test (VNT) and pseudotyped, luciferase-expressing vesicular stomatitis virus ΔG (VSVΔG) (35), we investigated the altered antigenicity of our GOF H mutants. Applying a panel of PPRV-specific (Fig. 9A) and MeV-specific (Fig. 9B) goat and human sera, respectively, we found a more variable neutralization response in human sera (Fig. 9B). In particular, certain human sera failed to equivalently cross-neutralize the GOF R191P pseudotypes (Fig. 9C), indicating that (i) the RBD represents an important cross-protective epitope, in line with existing knowledge about nAb epitopes within the MeV RBD (36, 37), and (ii) cross-protection in certain sera may be reliant on a limited pool of antibodies targeting only this epitope.

## DISCUSSION

Morbilliviruses are generally considered to have high host specificity, with disease manifestations being restricted to a narrow range of hosts, e.g., MeV in humans. Nevertheless, there is clear evidence that these viruses have jumped species into new hosts in the past (summarized by Nambulli et al. [38]). Indeed, recent CDV outbreaks in cynomolgus monkeys in China (39) and Japan (40) continue to illustrate the zoonotic potential of these viruses. The ability of morbilliviruses to use noncognate receptors functionally underpins this zoonotic potential. This is especially true for SLAMF1—the primary entry receptor—since its use is considered vital for establishing infection in the host (12). Supporting this, Sakai et al. demonstrated that a CDV from an isolate taken from a moribund monkey (CYN07-dV) was able to efficiently use utilize maSLAM to enter cells (41). Further investigations revealed that although CYN07-dV could not efficiently use hSLAM, repeated passage of this virus in Vero-hSLAM cells was sufficient to select GOF mutants, with a single P541S mutation in CDV H (corresponding to residue P545 in the RBD of MeV H) being demonstrably important (41). Similarly to residue 191, identified here as important in determining virus host range, this residue also appears to vary between morbilliviruses in a species-specific manner (Fig. 2A). Further evidence for the importance of these critical residues is provided by sequence analysis of RPV viruses adapted to rabbits—where a P191S mutation was observed (Fig. 2A). In addition, related amino acid changes, lying in close proximity to the CDV H RBD (position 549, corresponding to 553 in MeV) (Fig. 2A), have also been implicated in adaptation to varied carnivore hosts (42) and, in a study separate from those described above, hSLAM (D540G in CDV, corresponding to residue 544 in MeV) (Fig. 2A) (19). A key feature of these amino acids is that they lie in or in close proximity to the RBD of H. MeV residues equivalent to CDV P541 and D540 lie at the edge of the region of hydrophobic interactions between H and SLAMF1 termed “site 4” by Hashiguchi et al. (24). A report of a role of β-propellers 4 and 5 (in which site 4 sits) of the H head domain in SLAM binding predates the structural study by Hashiguchi, and there are considerable published mutagenesis data to support the idea of their importance in receptor interactions and host range (14). However, the structure of the MeV H-SLAM complex illustrated the key importance of additional residues in β-propeller 6 that make up the complete RBD, in particular, P191 to R195 in MeV H. This is essential to the formation of site 3 (Fig. 2)—an intermolecular β-sheet assembled by the polypeptide backbones of H 191 to 195 and SLAM 127 to 131. This region was not probed in earlier mutagenesis studies (14); however, we have now shown that amino acid changes in this region play a role in determining host range. Significantly, a recent study using a SLAM-blind, recombinant CDV mutant showed that partial reversion in a ferret model of disease was associated with compensatory mutations in the RBD, including the mutation T192A within site 3 (43). Using our functional assays of particle entry and cell-cell fusion, it is difficult to assess the exact biochemical nature of the altered interactions between H and SLAMF1. The most likely explanation is an alteration to protein-binding affinity; however, more-subtle effects on the ability of H to trigger F-mediated fusion (its fusion helper function) or on the relative levels of stability of H dimers and tetramers are also possible, especially given the close proximity of position 191 to the H stalk. While we did assess the relative levels of stability of the mutants (Fig. 3B and 5C), these issues are best addressed using direct protein-binding assays, the subject of ongoing investigations in our laboratory.

Separately, we demonstrated that changes within the HBS on SLAM can also have significant functional consequences for viral glycoprotein activity (Fig. 6 and 8). Using sequencing and structural modeling of nonhuman SLAMF1, Ohishi et al. identified a number of H residues (63, 66, 68, 72, 84, 119, 121, and 130) that are potentially important in determining host range (44). A number of these residues (63, 72, 119, and 130) were subsequently shown to lie directly within the SLAMF1 HBS (24), confirming their likely importance. These conclusions are now supported by our findings that modifications to these residues, in particular, L119 and R130, in hSLAM altered the host specificity of H interactions (Fig. 6 and 8, respectively).

Given the universal usage of SLAMF1 as an entry receptor for morbilliviruses and the relative similarities of the RBD of H and the HBS of SLAMF1 (Fig. 2A and 6A) at this interface, it is perhaps not surprising that specific amino acids appear critical in determining virus host range. The recent observation that the morbillivirus RBD (which overlaps for both SLAMF1 and Nectin-4 binding) is also a dominant and conserved neutralizing antibody epitope has helped to explain both the monoserotypic nature of morbilliviruses and the success of live attenuated vaccines (37). Our observation that changes to the RBD also affected antibody-mediated neutralization (Fig. 9) supports this conclusion and further indicates that, to some degree, this region may also represent an essential epitope for cross-protective neutralizing antibody binding.

The proposition that receptor tropism can inform studies on the zoonotic potential of paramyxoviruses represents an emerging area of interest (45, 46). As discussed by Zeltina et al. (46), the morbilliviruses and henipaviruses are especially interesting in this context because they bind proteinaceous receptors (SLAMF1 and Nectin-4 or EphrinB2 and EphrinB3, respectively)—with viral affinity for these proteins potentially determining host range. In drawing conclusions on morbillivirus host range, those authors relied on analysis of the overall variation of the RBD; however, by focusing separately on the four motifs that constitute this domain, we were able to identify a single site relevant to the host range, representing a hypothesis that was confirmed in our functional study and through analysis of nsSNPs within human SLAMF1. Our approach highlights the need for in-depth analysis of the structural interface between the attachment protein and the receptor and indicates that the morbillivirus host range might be determined by only a small number of amino acids within the entirety of the RBD. Whether this applies to more distantly related viruses, such as feline morbillivirus (Fig. 1A) or the recently identified morbilliviruses of bats and rodents (47), remains to be determined.

**FIG 10:**
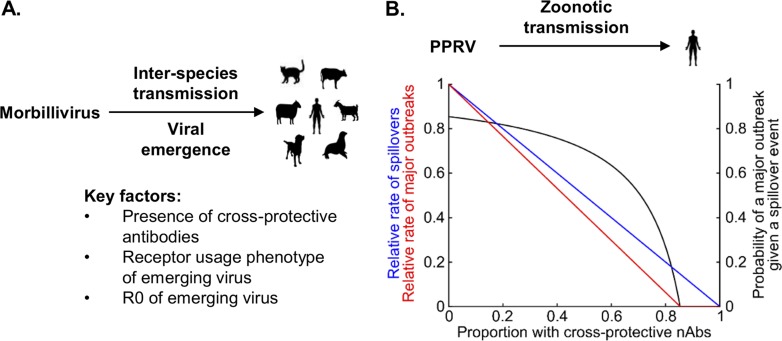
PPRV has zoonotic potential in human populations. (A) Factors influencing the emergence of morbilliviruses in atypical hosts. (B) The chance of PPRV emergence in humans. Blue line, the rate of spillovers of a variant of PPRV, capable of human-to-human transmission, into a human population in which a fraction *n* of individuals have cross-protective nAbs, relative to the equivalent rate in a population where no individuals have cross-protective nAbs [*z*(*n*)*z*(0)]; black line, following a single spillover event, the probability of a major outbreak driven by human-to-human transmission [*p*(*n*)]; red line, relative rates of major outbreaks in human populations, compared to a population where no individuals have cross-protective nAbs [*m*(*n*)*m*(0)]. Refer to Materials and Methods for detailed formulas. We assumed that a variant of PPRV capable of human-to-human transmission would have an *R*_0_ value of 6.85 in human populations with no cross-protective nAbs. This represents the estimated basic reproduction number of PPRV in Afghan (Bulkhi) sheep in Pakistan (60).

While there is no clear indication that enhanced entry alone is sufficient to confer a pathogenic phenotype to PPRV in humans, our minigenome experiments indicate that production of nascent particles in virus-infected human cells is likely. However, acquisition of a novel receptor usage phenotype might not immediately relate to pathogenesis in the host. Tellingly, the in-host reversion of receptor usage by a SLAM-blind CDV in ferrets did not restore virulence *in vivo*, even in a permissive host (43), perhaps because there was only partial reversion in SLAM binding. Separately, it was found that transgenic mice expressing hSLAM do not fully reconstitute a natural MeV infection when challenged (48). Other factors beyond entry, e.g., the efficiency of innate immune antagonism by the viral accessory proteins C and V, which are known to antagonize the host’s interferon response and may be species specific in their mechanism of action (49), are also likely to be important in the development of pathogenic and/or transmission phenotypes. However, since adaptation to a new host (through mutation and selection mechanisms) requires genomic replication inside infected cells, we would maintain that entry is the most important barrier to overcome, at least initially. This highlights the importance of both receptor usage and cross-protective antibodies in morbillivirus zoonotic transmission events, conclusions summarized in a simple model of morbillivirus emergence (Fig. 10A). Here, the probability of a major outbreak in atypical host populations is assumed to be dependent on receptor usage phenotypes and on the proportion of the population with cross-protective antibodies, as well as on the basic rate of reproduction (reproduction number) of the emergent virus. Applying this model to potential PPRV emergence in humans using a previously established *R*_0_ for this virus (Fig. 10B) highlights the potential need for continued vaccination in human populations. Similar patterns are likely to be relevant across the spectrum of morbillivirus hosts, particularly in cattle which no longer have high levels of immunity following the eradication of RPV and the cessation of vaccination.

While there are examples of single amino acid changes in H conferring enhanced tropism to human receptors, e.g., CDV (19), these events were identified using classical GOF experiments. Our study highlighted how it is possible to identify pathogens with zoonotic potential and GOF variants by structure-guided biochemical investigations. Using these “alternative” GOF approaches, we have demonstrated that receptor usage and cross-protective nAbs are important and distinct barriers to morbillivirus zoonotic transmission whose removal could have serious consequences for the ecological relationship between these viruses and their hosts, a finding that is especially significant given the ongoing eradication campaigns for PPRV and MeV, as well as the recent eradication of RPV. In addition, in light of our findings with respect to cross-neutralizing anti-MeV nAbs, we encourage epidemiological surveillance of mutations in this region, akin to the active surveillance programs for influenza virus (50). The 1986 isolation of a K191-encoding PPRV in the United Arab Emirates (accession number KJ867545) highlights the potential for natural variation at this position and supports the continued vaccination of humans and goats alike to both maintain high herd immunity levels and prevent emergence of PPRV in human populations.

## MATERIALS AND METHODS

### Plasmids and mutagenesis.

MeV F and H open reading frames (ORFs) were amplified from a patient isolate of MeV from Dublin, Ireland (51), following RT-PCR performed on RNA from infected Vero hSLAM cells. PPRV constructs were amplified from the PPRV reference strain (described previously [16]; AJ849636, Turkey 2000, field isolate, lineage IV). oSLAMF1 and hSLAMF1 were isolated from sheep and human monocytic cells, respectively. All constructs were cloned into pcDNA3.1 (Thermo Fisher). MeV H expression constructs were amplified to include an N-terminal hemagglutinin (HA) tag. Site-directed mutagenesis was confirmed by sequencing. The specific cloning strategies and primer sequences used are available upon request.

### Cell-cell fusion assays.

HEK293T effector cells were transfected (using Transit-X2 transfection reagent [Mirus] per the manufacturer’s instructions) with 500 ng each of MeV or PPRV F and H expression constructs and 250 ng of the 1-to-7 fragment of recombinant luciferase-green fluorescent protein (rLuc-GFP) (52). Separately, target cells were transfected with 1 μg of various SLAMF1 expression constructs, as well as with 250 ng of the 8-to-11 fragment of rLuc-GFP. At 48 h posttransfection, effector and target cells were washed, counted, and cocultured at a ratio of 1:1 in white-walled 96-well plates to a final density of 1 × 10^5^ cells per well. At 16 to 24 h postcoculture, the *Renilla* luciferase activity in fused cells was measured (in a Promega GloMax multimode plate reader) by removing the media and adding 2 μg/ml of cell-permeative coelenterazine 400a (Biotium) diluted in phosphate-buffered saline (PBS). Apart from the assay whose results are presented in Fig. 1D and E, all fusion assays were performed with a cognate F protein from the same viral strain. For Fig. 1D and E, the F protein from the Senegal lineage 1 strain was used with PPRV Senegal, Benin, Kenya, and Ethiopia H. PPRV F proteins are highly conserved, with >95% identity between strains (data not shown). Four or more coculturing biological replicates were performed for each biological condition. All experiments were performed a minimum of three times.

### Pseudotyped viruses.

Morbillivirus F and H expression constructs with truncated cytoplasmic tails (ΔF/ΔH [30 and 24 aa, respectively]) were cloned and used for pseudotype production and quantitative entry experiments as described previously (30). Briefly, HEK293T cells were plated for pseudotype production at a density of 7.5 × 10^5^ cells per well in 6-well dishes and were transfected the following day with 3.5 µg of each of the pcDNA3.1-ΔF/ΔH constructs, as well as with 1.5 µg of p8.91 (encoding HIV-1 *gag-pol*) and 1 µg of CSFLW (the luciferase reporter expressing the lentivirus backbone). Matched cognate combinations of F and H from PPRV and MeV strains were used in all assays. Supernatants containing pseudotyped viruses were harvested at 72 h posttransfection, clarified by centrifugation, and frozen at −80°C. Target cells were plated at a density of 2 × 10^4^ cells per well in 96-well dishes 1 day prior to transduction/infection for 72 h. Firefly luciferase activity in these cells was assayed using a luciferase assay system (Promega) according to the manufacturer’s instructions and a Promega GloMax multimode plate reader.

### Surrogate VNTs.

To prepare VSVΔG*luc* pseudotypes, HEK293T cells were transfected with the H and F expression vectors from the respective viruses, followed by superinfection with VSVΔG*luc* (VSVG) as described previously (53, 54). Supernatants were harvested 48 h postinfection, divided into aliquots, and frozen at −80°C. The titer of each viral pseudotype stock was estimated by preparing serial dilutions in triplicate and plating onto 293 canine SLAM cells followed by incubation for 48 to 72 h at 37°C, at which time luciferase substrate was added (steadylite plus; Perkin Elmer) and the signal analyzed on a MicroBeta 1450 Jet luminometer (Perkin Elmer). Canine SLAM was used because neither MeV nor PPRV appears to be restricted by this receptor (data not shown). The viral titer (50% tissue culture infectious dose [TCID_50_]) was calculated using the Spearman-Kärber formula. To measure virus neutralization, 4-fold serum dilutions ranging from 1:8 to 1:32,768 were prepared in triplicate and added to 293 canine SLAM cells in 96-well white flat-bottomed plates, followed by 2.5 × 10^3^ TCID_50_ of VSVΔG(F&H) pseudotype. Plates were incubated for 48 h at 37°C, at which time luciferase assays were performed. Antibody titers were calculated by interpolating the point at which there was a 90% reduction in luciferase activity (90% neutralization or 90% inhibitory concentration [EC_90_]).

### Minigenome assays.

Minigenome assays were performed as described previously (55) using human A549 cells and a PPRV Turkey 2000 minigenome with a Gaussia luciferase reporter gene.

### Protein biochemistry.

All protein samples were prepared in 1× radioimmunoprecipitation assay (RIPA) buffer containing protease inhibitors (Thermo Fisher). Briefly, the existing growth medium was removed and cells were washed in phosphate-buffered saline (PBS) before being pelleted by centrifugation. Pelleted cells were then resuspended in 1× RIPA buffer and left on ice for 10 min before repeated centrifugation was performed at high speed (16,000 × *g*) for a further 10 min at 4°C. Protein lysate-containing supernatants were then stored at −20°C until required. Samples for Western blotting were analyzed by SDS-PAGE, semidry polyvinylidene difluoride (PVDF)-based transfer, and blotting in Tris-buffered saline (TBS)-Tween containing 5% (wt/vol) milk powder. All primary antibodies were incubated overnight at 4°C. Western blotting was performed using the following antibodies: anti-morbillivirus/MeV hemagglutinin (cytoplasmic tail) (H-cyt) (rabbit polyclonal; a gift from R. Cattaneo [56]) (1:1,000), anti-FLAG (9A3; Cell Signaling [CS]) (1:1,000), anti-GAPDH (anti-glyceraldehyde-3-phosphate dehydrogenase) (14C10; CS) (1:1,000), anti-HA (C29F4; CS) (1:1,000), anti-HIS (CS) (1:1,000), SLAM (N-19), Santa Cruz sc-1334, and standard horseradish peroxidase (HRP)-linked secondary antibodies (CS).

### Probability of a major outbreak.

Mathematical modeling was used to assess the probability of a PPRV outbreak in humans. Consider a cross-protected population where a proportion *n* of individuals are completely protected by cross-protective nAbs and the rest have no protection. The rate of spillover events of a PPRV variant, capable of replication within humans, into this population can be written as *z*(*n*) = *z*(0)(1 − *n*), where *z*(0) is the rate of spillover into a fully susceptible population in which no individuals are cross protected. Once a spillover has occurred, the probably of a major outbreak is given by $p(n)=1-\frac{1}{\left(1\;-\;n\right)R_{0}}$ if (1 − *n*)*R*_0_ = >1 or by *p*(*n*) = 0 otherwise (57), where *R*_0_ is the basic reproduction number of the PPRV variant in a fully susceptible population where no individuals are cross protected (Fig. 10B, black line). Multiplying these two quantities gives the expected rate of major outbreaks as follows: *m*(*n*) = *z*(*n*)*p*(*n*). Since *z*(0) is an unknown quantity, we determined the relative rates of spillover events and major outbreaks in cross-protected populations and fully susceptible populations, giving *z*(*n*)/*z*(0) = (1 − *n*) and $m(n)/m(0)=1-\frac{R_{0}\left(1\;-\;n\right)-1}{R_{0}\;-1}$, respectively (Fig. 10B, blue and red lines).

### Bioinformatics and modeling.

Comparison of amino acid sequences was performed using the Vector Nti package (Thermo Fisher) and AlignX embedded software as well as the Weblogo online server (58). Information regarding nsSNPs within human SLAMF1 was obtained from the ExAC database (32), while ovine SLAMF1 variations were mapped as described previously (31). Models of the PPRV H and ovine SLAM structures were generated using the I-TASSER Web server (59) and the MeV H and maSLAM structures from PDB 3ALX (24) as templates, respectively. Models of oSLAMF1:MeV H and PPRV H:maSLAMF1 complexes were generated by superposing models of oSLAM and PPRV H onto maSLAM and MeV H, respectively, from the high-resolution structure of the complex (PDB 3ALX) (24). Molecular images were generated using PyMOL (Schrodinger LLC).

## Supplementary Material

Supplemental file 1
